# Implantation of 3D-Printed Patient-Specific Aneurysm Models into Cadaveric Specimens: A New Training Paradigm to Allow for Improvements in Cerebrovascular Surgery and Research

**DOI:** 10.1155/2015/939387

**Published:** 2015-10-11

**Authors:** Arnau Benet, Julio Plata-Bello, Adib A. Abla, Gabriel Acevedo-Bolton, David Saloner, Michael T. Lawton

**Affiliations:** ^1^Skull Base and Cerebrovascular Laboratory, Department of Neurosurgery, University of California San Francisco, San Francisco, CA 94143, USA; ^2^Department of Neurosurgery, Hospital Universitario de Canarias, 38320 Tenerife, Spain; ^3^Department of Neurosurgery, University of California San Francisco, San Francisco, CA 94143, USA; ^4^Center for Cerebrovascular Research, University of California San Francisco, San Francisco, CA 94143, USA

## Abstract

*Aim*. To evaluate the feasibility of implanting 3D-printed brain aneurysm model in human cadavers and to assess their utility in neurosurgical research, complex case management/planning, and operative training. *Methods*. Two 3D-printed aneurysm models, basilar apex and middle cerebral artery, were generated and implanted in four cadaveric specimens. The aneurysms were implanted at the same anatomical region as the modeled patient. Pterional and orbitozygomatic approaches were done on each specimen. The aneurysm implant, manipulation capabilities, and surgical clipping were evaluated. *Results*. The 3D aneurysm models were successfully implanted to the cadaveric specimens' arterial circulation in all cases. The features of the neck in terms of flexibility and its relationship with other arterial branches allowed for the practice of surgical maneuvering characteristic to aneurysm clipping. Furthermore, the relationship of the aneurysm dome with the surrounding structures allowed for better understanding of the aneurysmal local mass effect. Noticeably, all of these observations were done in a realistic environment provided by our customized embalming model for neurosurgical simulation. *Conclusion*. 3D aneurysms models implanted in cadaveric specimens may represent an untapped training method for replicating clip technique; for practicing certain approaches to aneurysms specific to a particular patient; and for improving neurosurgical research.

## 1. Introduction

Cerebrovascular surgery is challenging and requires refined technical skills and thorough knowledge of both brain vasculature and skull base approaches. Progressive technical advances and good outcomes in patients with aneurysms treated with endovascular techniques have modified surgical indications for aneurysm clipping [1, 2]. At present, very complex and challenging cases are presented more often to cerebrovascular surgeons practicing at tertiary centers.

Research and training are essential for safe surgical treatment of challenging cerebrovascular lesions. Surgical research is critical for designing better approaches for the treatment of difficult-to-reach aneurysms (e.g., basilar apex aneurysms) and arteriovascular malformations (AVMs). Also, educational models for cerebrovascular surgery are very important to prepare neurosurgeons in managing challenging lesions early in their careers.

At present, there are two main models for surgical training that complement surgical experience: augmented-reality computer models and cadaveric surgical simulation. Although very promising, the augmented-reality computerized models do not provide a realistic surgical environment due to many technological limitations (e.g., lack of brain manipulation or realistic surgical feedback). Cadaveric surgical simulation is one of the best complements to surgical experience as it allows very realistic surgical scenarios to be simulated. Our group has recently published a customized embalming formula for neurosurgical research that preserved the natural physical features of the brain, most importantly retraction qualities, therefore providing a very realistic surgical environment [3]. There are several reports on neurosurgical educational models using human cadavers in the literature [4–8]. Aboud et al. designed a model to simulate human blood flow in disease-free human cadavers for neurosurgical training [7]. However, none of the models described in the literature include the simulation of cerebrovascular surgical scenarios in cadavers, using aneurysm models of lesions from actual patients.

We propose a model of cerebrovascular surgical simulation that includes, for the first time, the implantation of 3D-printed replicas from real cerebrovascular lesions in human cadavers. Using 3D-printed aneurysm models obtained from patient's radiological studies, we recreated several surgical scenarios to investigate the potential use of this model for neurosurgical research and training. In this report, we describe our method and provide initial results on the application of this model to basilar apex and middle cerebral artery aneurysms.

## 2. Materials and Methods

In this model, we generated 3D-printed aneurysms from preoperative radiological studies of previously selected patients and implanted them into 2 human cadavers (4 specimens) prepared for surgical simulation. We also performed different approaches on each side of the specimens to evaluate the manipulation capabilities, visualization, and surgical exposure of the models and discuss their potential role in cerebrovascular research and education.

### 2.1. Preparation of Cadaveric Heads

The human heads were sectioned in the neck at the level of C7. The carotid and vertebral arteries as well as the jugular veins were dissected 1 cm cranially from the section level to allow cannulation. Tubing of different size was introduced into each vessel and ligated using 2-0 suture. Then, the vessels were profusely irrigated with saline solution until all blood and blood clots were cleared out from the vasculature. Next, a customized embalming solution [3] was manually injected through all cannulated vessels repeatedly and the specimen was stored for one week at room temperature. At this point, the specimens were divided into two groups: (a) cerebrovascular training or (b) surgical research. The criteria for selection were designed to assign those specimens with best quality to the surgical research group and included donors younger than 75 years old and minimal degeneration of the spinal cord at the edge of the neck section (which was used to infer the quality of the encephalon before dissection). For training purposes, and to simulate surgical bleeding, the specimens were connected to two liquid reservoirs inside pressure bags [7]. The tubing previously introduced in the arteries was connected to one reservoir containing red colored saline solution at variable arterial pressure (approximately 120–140 mmHg). Tubing from the jugular veins was connected to a reservoir containing dark blue saline solution at low pressure (40–60 mmHg). After several training sessions, the specimens in this group, already embalmed, were transferred to the research group for bypass investigation.

The specimens selected for research were prepared for latex injection to ease vessel identification during dissection and maintain the vasculature in optimal conditions for all research time. Red latex was injected to the common carotids and vertebral arteries bilaterally until the arterial system was completely filled. Then, dark blue latex was injected to both jugular veins. Next, all tubing was clamped and the specimens were stored immersed in embalming solution for 72 hours to allow the latex to cure.

### 2.2. Aneurysm Model Production

The patient database of the senior author (M. T. Lawton) was searched for patient selection. For research purposes, our selection criteria include the following: (1) an aneurysm is considered suitable for surgical treatment (broad neck, complex anatomy, and perforators arising from the neck/dome), but surgical access is deemed complex or no proximal control could be guaranteed; (2) surgical treatment was suboptimal (incomplete closure, perforator entrapment); (3) poor patient outcome is related to the surgical approach; (4) intraoperative aneurysm rupture is present. For training purposes, we use the following inclusion criteria: (1) aneurysm shape is best suited for surgical treatment; (2) aneurysm is accessible from one approach; (3) proximal control of the aneurysm is feasible. After patient selection, the radiological studies were anonymized and transferred to the center for cerebrovascular research for aneurysm model reconstruction.

**Figure 1:**
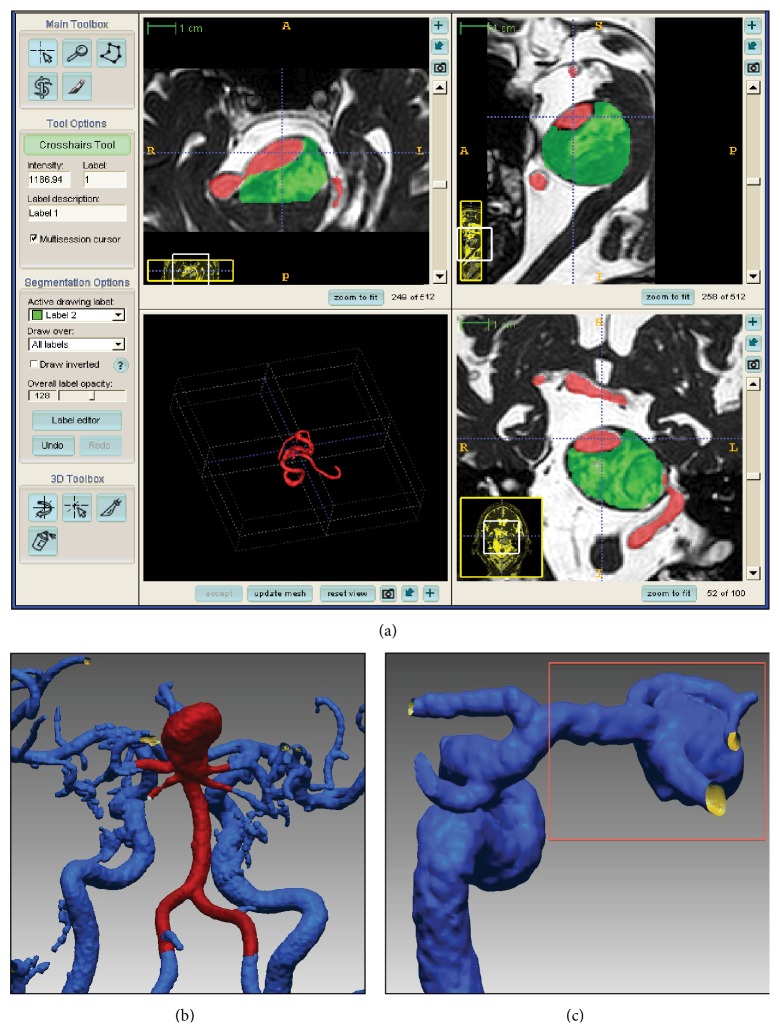
Radiological reconstruction of the 3D aneurysm model from a selected patient. Using dedicated software, the aneurysm thrombus (green) and lumen (red) were identified and labeled in each slice of the magnetic resonance of the selected patient (a). The software-generated vascular model was obtained for a patient with a basilar apex (b) and middle cerebral artery (c) aneurysm and the zone of interest was selected and cropped for printing (brown square).

Part of our preoperative radiological protocol for patients with cerebral aneurysms includes contrast-enhanced magnetic resonance angiography (CE-MRA) (TR = 5.5 ms, TE = 1.8 ms, 0.5 × 0.5 × 1.2 mm) and a balanced fast field echo sequence (TR = 5.2 ms, TE = 1.8 ms, 0.5 × 0.5 × 1.2 mm), which were acquired on an* Intera* 1.5T scanner (Phillips Healthcare, Best, Netherlands). These studies are always set in the same volume and orientation to visualize the vessel lumen and surrounding thrombus. The CE-MRA dataset was loaded into a dedicated software package [9] (*ITK-SNAP*, http://www.itksnap.org/) for segmentation (Figure 1). The software generated an accurate real-sized surface of the lumen, which was then saved. The balanced fast field echo dataset was then loaded into a new window along with the CE-MRA surface. The intraluminal thrombus dimension was labeled manually while the CE-MRA surface served as a marker for the lumen position (Figure 1(a)). The combined surfaces were saved and exported to a 3D volume file (Figures 1(b) and 1(c)), which was printed in 3D (Fathom, Oakland, CA) using rubber-like material (tangoBlackPlus) with shore hardness *A* = 25.

### 2.3. Aneurysm Implantation

The 3D aneurysm model was implanted in the specimens at the same anatomical region where it was originally located in the patient. An open craniotomy was performed in the specimen, and the implantation zone was exposed using standard surgical techniques: dural opening, arachnoid dissection, cerebrospinal fluid suction, and brain retraction. A surgical microscope (Carl Zeiss Pentero) was used for intradural dissection. The implantation zone was carefully exposed with minimal dissection of the arachnoid cisterns. The aneurysm model was implanted to the vasculature using cyanoacrylate (Loctite) with anastomosis of the surrounding vessels to create a vascular model similar to that of the actual patient. In the research group, the branches of the 3D model were glued to the latex in the lumen of the cadaver vasculature. In contrast, the branches of the 3D models implanted in the training group were anastomosed to the cadaver vessels using 8-0 sutures. This was possible because the lumen of the vessels in the training group was hollow, and the 8-0 needle could be passed through the 3D model's branches without deforming. To implant giant aneurysms that generate mass effect to the surrounding tissue, a Foley catheter was inserted first by us and inflated progressively at the implantation side to create anatomical deformity related to mass effect and obtain the room required to implant the aneurysm model.

## 3. Results

**Figure 2:**
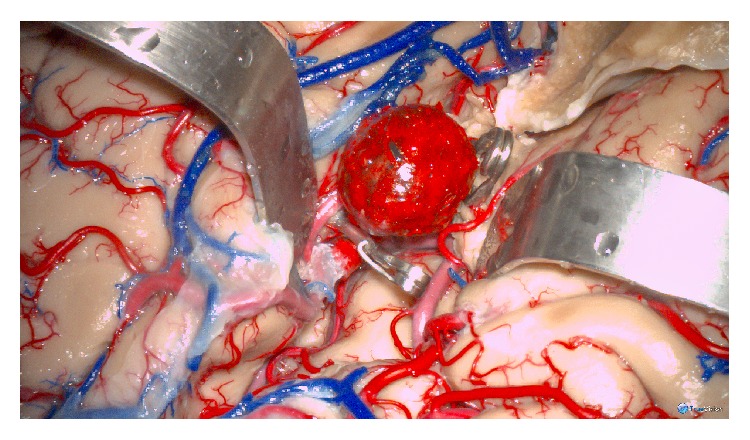
Surgical simulation photograph of a 3D-printed aneurysm model implanted in a left-side opercular artery. The sylvian fissure was completely split and the opercular arteries (M2) were exposed. The aneurysm was implanted into the proximal segment of one opercular artery. Both temporal clips and permanent clipping of the aneurysm neck were trained during the surgical simulation.

Basilar apex and middle cerebral artery aneurysm models were created for evaluation. We selected these aneurysms from the senior surgeon's database because they represent two frequent lesions. We also selected the basilar apex model to illustrate a lesion that requires further surgical research [10, 11]. A pterional approach was performed. The sylvian fissure was carefully dissected as previously described [12] and the deep sylvian cistern was exposed. A Foley catheter was inserted into the anterior half of the deep sylvian cistern and inflated 5 mL to create mass effect. The procedure was resumed the next day to allow full expansion of the sylvian fissure. Following this, the aneurysm replica was glued to the target middle cerebral artery, proximal M2 (Figure 2). After clipping techniques and possible bypass anastomoses were tried, the aneurysm was detached from the specimen.

**Figure 3:**
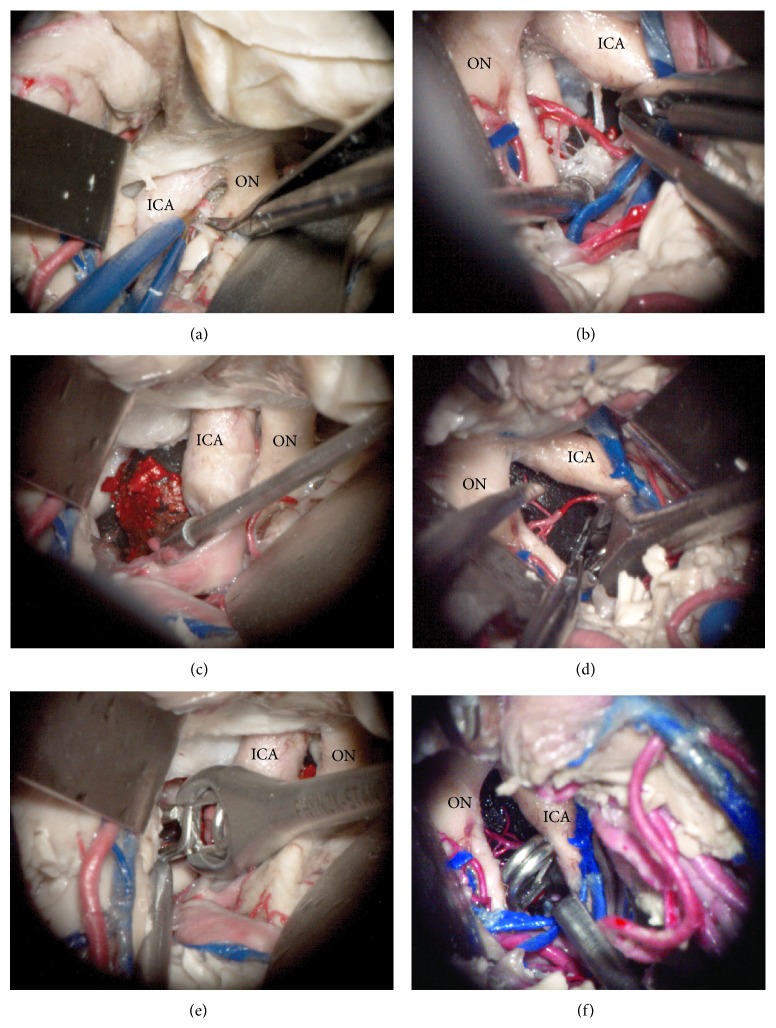
Microscopic photography of left (a, c, and e) and right (b, d, and f) surgical simulation experiments for implanting and clipping a basilar tip aneurysm. After a wide sylvian fissure split, the left carotid cistern was dissected to access the interpeduncular fossa (a). The 3D aneurysm model was successfully implanted through the oculomotor triangle and positioned to match the target anatomy, basilar apex (c). Next, an angled aneurysm clip was introduced and positioned across the aneurysm neck in the basilar apex (e). Then, the right transsylvian approach was performed with the aneurysm model already implanted. The arachnoid dissection and perforator arteries manipulation were affected by the aneurysm mass effect (b). The dome of the previously implanted aneurysm was mobilized to identify the aneurysm neck (d) and an angled clip was applied to the basilar apex aneurysm neck (f). ICA: internal carotid artery, ON: optic nerve.

Next, the orbitozygomatic (OZ) piece was taken [13]. The frontal and temporal opercula were retracted and the carotid cistern was dissected. The temporal pole was untethered by dividing the vein to the sphenoparietal sinus and cutting arachnoid adhesions to the middle fossa. Next, the temporal lobe was retracted posteriorly and laterally to expose the interpeduncular cistern through the carotid-oculomotor triangle. The precommunicating segment of the posterior cerebral artery (P1) was followed proximally to the basilar apex with extreme care to preserve the thalamoperforators. At this point, the Foley catheter was inserted and inflated 3 mL to create room for implantation and left under pressure overnight. Finally, the basilar apex aneurysm model was implanted (Figures 3(a), 3(c), and 3(e)). A contralateral OZ was performed to simulate the surgical clipping of the basilar apex model (Figures 3(b), 3(d), and 3(f)). One permanent clip was used during the clipping simulation experiment to obliterate the aneurysm in the basilar apex model.

The 3D aneurysm models were successfully implanted to the specimen arterial circulation in all cases. The flexibility of the neck and branches of the aneurysm model allowed successful clipping and manipulation similar to those of a partially thrombosed aneurysm. The dome was also stiff enough to produce mass effect to the surrounding structures and preserve the original size and shape.

Four surgical approaches were successfully performed in each specimen (Table 1). The implanted aneurysm model maintained the original position consistently in all simulations. A navigation probe was successfully passed through the recreated surgical exposure, proving that morphometric comparative studies applied to neurosurgical simulation are possible using a real aneurysm model.

### 3.1. Model Applications in Cerebrovascular Surgical Simulation

This method provided three main advantages for cerebrovascular surgical training. (1) The aneurysm implantation technique required meticulous dissection of disease-free anatomy, which provided the prospect of learning the surgical anatomy of the implant zone before being distorted by the aneurysm model. (2) The mass effect created by the Foley catheter and the introduction of the aneurysm model into the normal vasculature offered a unique opportunity to understand the patients' neurological disease state caused by tissue compression and identify the critical structures beyond the aneurysm dome that must be preserved during aneurysm dissection including vital arterial perforators. (3) An aneurysm replica implanted into a human cadaver prepared for bleeding simulation provided an optimal surgical simulation scenario to learn and train dissection techniques and maneuvers to safely clip and bypass real aneurysms.

The permanent implantation of an exact replica of a patient aneurysm provided unlimited time to conduct research; cadaver features remained constant throughout the experiments. Four surgical approaches were successfully performed in each specimen. The implanted aneurysm model maintained the original position consistently in all simulations. A navigation probe was successfully passed through the recreated surgical exposure and to the implanted aneurysm, demonstrating that future morphometric comparative studies could be carried out using a real aneurysm model.

## 4. Discussion

The development of the neuroendovascular field has brought new perspectives to vascular neurosurgery. On one hand, endovascular procedures are becoming safer and more applicable to many types of aneurysms [14], increasing the rate of aneurysms treated with this method [1]. On the other hand, in many centers, the cases that are considered for surgery are the most complex ones, whose endovascular treatments have failed or are unsuitable [1, 15]. Because the number of aneurysms considered for surgery is decreasing and becoming limited to the most complex ones, the degree of expertise required for its treatment is becoming harder to achieve. In fact, the current caseload has been reportedly limited even for simple cerebral aneurysms [16]. This volume-outcome effect has been reinforced by larger regional and national databases [17]. Due to this, new methods have to be considered for effective microsurgical training and simulation to prepare future generations for surgical aneurysm clipping [18].

This work presents a novel and promising anatomical model for the study and training of brain aneurysm surgery. In this model, the neck and branches of the aneurysms showed flexibility similar to that of the living human. The rigidity of the aneurysm dome was optimal to simulate compression to the surrounding brain structures. Although tested in only four specimens, the 3D aneurysm model presented here proves feasible for implantation in the embalmed cadaver and optimally emulates the features of an aneurysm.

Other 3D aneurysm models have been previously reported. D'Urso et al. (1999) created biomodels of cerebrovascular lesions and stressed their use for complex case preparation or when radiological data is unsatisfactory [19]. Similarly, Wurm et al. (2004) reported the utility of solid plastic 3D models to better understand the anatomy of aneurysms, further illustrating the superiority of 3D models to other imaging methods [20]. In 2009, Kimura et al. used a 3D aneurysm model effectively for preoperative simulation and surgical training [18]. The model by Kimura et al. successfully allowed the practice of clipping due to its softness and elastic features, exceeding other previous 3D models in that it allowed for surgical training. In some cases, they also developed a hard 3D model for representing the skull base and the rest of the vasculature [18]. Similar results were subsequently reported with a 3D model proposed by Mashiko et al. (2011) [21]. Like the previous models, the ability to estimate the clip(s) position, size, and shape preoperatively when using our model allows one to avoid the occlusion of parent vessels and to predict the best combination of clips to obliterate the aneurysm. However, when the 3D model is implanted in a cadaveric head, there are more anatomic-related aspects that can be identified and analyzed (e.g., angle of attack, clip fenestration, and limited exposure). Thus, we have gone a step further in the recreation of an aneurysm operative environment in that the previous aneurysm models were not implanted in a naturalistic environment.

Although 3D radiological reconstructions have notably improved the display of brain aneurysms and have provided an important tool for neurosurgeons, there are certain cases where this information might be insufficient to properly conceive certain features of an aneurysm such as intraluminal thrombus. Furthermore, the 3D reconstruction does not identify the relationships between the surrounding brain structures and the aneurysm, which are essential in vascular surgery. In the most complex cases, the creation of a 3D 1 : 1 replica of the patient's aneurysm and its subsequent implantation in a cadaveric specimen may assist the surgeon in determining how to attack the aneurysm by selecting the best positioning and approach. Bearing this in mind, standard craniotomies may be modified to achieve better visualization and control of the lesion and to avoid potential damages to other brain structures secondary to manipulation. As previously stated, using this sort of 3D model allows one to predict the type and number of clips as well as the way they should be applied for best results [18]. In fact, the neurosurgeon can recreate the surgical procedure in the laboratory and modify his/her previous strategy (originally based on radiological reconstructions) as he/she realizes that certain modifications can improve the feasibility of the surgical procedure and, eventually, the final outcome of the patient. Therefore, the model proposed in this report allows for a thorough study and optimal design of a particular case, which could be considered a form of personalized surgical therapy. Although the most important limitation of this procedure is that the bony anatomy in the cadaver would not be specific to the patient who needs treatment, this model provides the most realistic method to test the different surgical options and weigh their differential benefits and risks for a particular type of aneurysm. In other words, the 3D aneurysm model implanted in a cadaveric specimen would prepare the neurosurgeon to the “surgical battle,” choosing the best strategy to defeat a specific and carefully assessed enemy with the least collateral damages.

The impact that this cadaveric simulation model may have in the training of resident neurosurgeons and inexperienced neurosurgeons should also be taken into consideration. As previously stated, endovascular success is leading to limited exposure to aneurysm surgery during residency training [22]. In centers with large experience and comprehensive cerebrovascular teams, this situation exposes residents to challenging cases early in their training, without adequate experience in the simplest cases. However, in centers without a strong cerebrovascular caseload, residents may not be confronted with enough cases to achieve self-confidence and skills in the management of aneurysms to safely transit them to unsupervised practice. Several studies have demonstrated a negative correlation between operative outcomes and the experience of a surgeon [23–25]. Therefore, there is a greater need for developing measures that ensure training is tailored to maintain proficiency in cerebral aneurysm surgery [22].

3D virtual reality environments have been created as a tool for neurosurgical trainees to learn surgical approaches to vascular lesions [26]. Although the advances in virtual reality computerized models and neuronavigation have been presented as potential contributors to training surgical skills [27], it is widely agreed that human cadaveric models are anatomically the most realistic ones [28].

With this in mind, the application of the 3D aneurysm implantation model presented here may help overcome decreased clinical exposure to simple aneurysm cases by including hands-on training in the most frequently surgically treated aneurysms. All the previous 3D aneurysm models reported have emphasized the potential utility of this biomodels for trainees [18–20, 29]. This educational feature has also been considered in the present work; as explained above, a 3D model for training purposes was created (see Section 2) and suitably implanted in a cadaveric specimen. Advanced residents and novel neurosurgeons could take advantage of this model and revise the approaches to the most frequent aneurysms stepwise in a more realistic environment. The repeated exposure and participation in operative procedures are considered essential in a surgical training program [30]. Training with cadaveric-implanted 3D models could lead to increased competence required to provide safe and efficient treatment to the most urgent and complex cases. In addition, this model might also be useful in objectively evaluating the acquisition of core competence and skills in cerebrovascular surgery.

Although the utility of 3D models may be accepted as useful tool for surgical planning and training, it should not be considered as a substitute of the active participation in real neurovascular surgeries. These models should be considered as tools for developing microsurgical abilities that can be later applied to real patients under supervision of an experienced surgeon. The use of these new educational tools, one day, may not only compensate for decreased microsurgical experience, but also improve neurovascular surgery clinical outcomes.

The models used in the present study were rigid and compact to represent both partially thrombosed aneurysms and the mass effect against the surrounding anatomy. However, the current printing technology allows for hollow-type vessel printing, which our team had successfully printed in the past. One of the benefits of 3D-printed aneurysms for teaching and training institutions is that they can be used multiple times and implanted in different locations with similar target vessel diameter, posterior inferior cerebellar artery and anterior inferior cerebellar artery.

Another potential application of the presented model is surgical research. 3D radiological models were developed to simulate the intra-aneurismal hemodynamic, which is associated with aneurysmal growth and whose appropriate evaluation is critical for endovascular procedures [31–34]. However, when surgery is the selected treatment, it is common to confront cases where the surgical approach is controversial (e.g., basilar tip aneurysms) or is not as standardized as others. Assessing the different approaches for controversial microsurgical management of complex aneurysms (aneurysms without a sole optimal approach) can provide scientific solutions to surgical dilemma. We are currently using this model to assess the available surgical approaches to several complex and controversial aneurysm cases by means of morphometric analysis (surgical freedom, surgical corridor, surgical exposure, and angles of attack) and comparative anatomical evaluation. This method represents an improvement in the field of neurosurgical research as it allows measurements and exposures to be made on an actual aneurysm (surgical target) in an aneurysm-related spatial distortion rather than in a random anatomical point.

Considering that this method adds to the cost of the conventional cadaveric training, it is essential to select appropriate cases for performing such 3D model implantations and eventually recreating the surgical conditions. Most simple 3D aneurysm models may be useful for residents training, and standard models can be generated to this purpose, which could lead to industrial production and lowering costs. Nevertheless, complex cases that typically have specific aneurysm features must be carefully selected. In this regard, large/giant size aneurysms are proven to be largely associated with the outcome and surgical difficulty [1]. Moreover, the location (being posterior circulation aneurysms of high complexity), previous coiling, and complex patterns of perforators might be other features that increase the complexity when surgically clipping an aneurysm. In all cases that present one or more of these complexities, the model presented in this report may be helpful for getting the best surgical result.

### 4.1. Study Limitations

At present, 3D printers are limited to models larger than 1 mm if plastic compounds are chosen. Therefore, patients with very small aneurysms cannot be reproduced using our model. The reproduction of the exact features of an aneurysm from a particular patient can be challenging and limited by the current resolution of the radiological studies. Perforator branches taking off from the aneurysm dome and/or its vicinity are not typically identifiable in an MRI. Thus, inability to reconstruct real aneurysm perforators is of technological nature and therefore an absolute limitation of the present. However, the constant resolution optimization in medical technology could potentially make perforators visible in routine patient neuroimaging and overcome this limitation. Therefore, legitimate reconstruction of aneurysm perforators is a future possibility. In the meantime, an approximation to realistic aneurysm perforator reconstruction could be based on postoperative photographic review and deliberate generation and rendering of the perforators in the model creation process.

Using postmortem models has inherent limitations. In this model, evaluation of both successful aneurysm clipping and preservation of surrounding perforators is limited to visual inspection. One of the obvious limitations of the present model is the lack of clinical deficits from perforator entrapment or sustained retraction. However, we see this limitation as one of the benefits of our model, which can serve as a harmless battleground for cerebrovascular innovation through practice. In the same way, cerebral “relaxation” with the use of intravenous mannitol and other strategies used in anesthesia to ease surgical maneuverability are not possible to recreate. However, we see this limitation as another benefit of our model as it forces new surgical strategies to be tested in a surgical scenario much more limited than the real procedure in patients. The reader should note that a customized embalming formula for neurosurgical simulation was used [3]. The specimens embalmed with our method provided significantly better retraction profiles than those treated with classic embalming solutions. Successful aneurysm implantation, especially of giant aneurysms, is highly dependent on brain manipulation. We routinely use the customized embalming solution for neurosurgical simulation and have not tried our 3D aneurysm implantation model in specimens embalmed with other solutions.

## 5. Conclusion

Cerebrovascular surgery is a demanding specialty that requires the acquisition of certain microsurgical skills for successfully clipping aneurysms. Shifting surgical trends brings to cerebrovascular surgery increasingly complex cases, which may require treatment by less experienced neurosurgeons. Our 3D aneurysm model represents a good surgical model for studying relative cerebrovascular anatomy, for confirming the aneurysm's relationship with other brain structures, and for identifying which surgical approaches are the most suitable for confronting certain aneurysm.

This model provides a substantial improvement in cerebrovascular surgical simulation. The application of this model in cerebrovascular research could provide an unparalleled opportunity to design better surgical options for patient-specific aneurysms in cadavers. This method allows surgical exposure similar to that in the real surgical procedure, replicating the constraints of particular surgical corridors in the setting of clip application and the relative surgical freedom to apply aneurysm clips between specific surgical corridors (on a cadaver) to certain patient-specific aneurysms.

It may serve as an indispensable tool for selecting the most suitable surgical approach in complex cases. The contribution of these models in operative training and neurosurgical research are now as important now as ever, given the continuous decline in case volume in complex cerebrovascular surgical cases available for resident training.

## Figures and Tables

**Table 1:** Surgical simulation experiments for aneurysm clipping in cadaver.

3D model	Approach for implantation	Side of implantation	Approach for clipping
Basilar tip	Orbitozygomatic	Left	Right orbitozygomatic
	Orbitozygomatic	Right	Left orbitozygomatic
	Orbitozygomatic	Left	Right orbitopterional
	Orbitozygomatic	Right	Left orbitopterional

Middle cerebral artery	Pterional	Left	Left pterional
	Minipterional	Right	Right minipterional
	Pterional	Left	Left pterional
	Pterional	Right	Right minipterional
